# The Impact of Local Economic Growth Target Setting on the Quality of Public Occupational Health: Evidence From Provincial and City Government Work Reports in China

**DOI:** 10.3389/fpubh.2021.769672

**Published:** 2021-10-25

**Authors:** Fan-Fan Wang, Wen-Jing Deng, Hao Cheng, Qing Gao, Zi-Wei Deng, Han-Cong Deng

**Affiliations:** ^1^School of Public Administration, South China University of Technology, Guangzhou, China; ^2^Department of Computer, Guangdong Electronic Commerce Technician College, Guangzhou, China; ^3^Key Laboratory of Environment Change and Resources Use in Beibu Gulf Ministry of Education, Nanning Normal University, Nanning, China; ^4^College of Economics and Management, Nanning Normal University, Nanning, China; ^5^Graduate School, Nanning Normal University, Nanning, China; ^6^School of Economics and Management, Guangxi Normal University, Guilin, China; ^7^School of Finance and Public Administration, Yunnan University of Finance and Economics, Kunming, China

**Keywords:** economic growth target setting, constraint, gap, government work reports, quantity of public occupational health

## Abstract

This article uses data from the government work reports of 31 provinces (autonomous regions and municipalities) in China and 21 cities in Guangdong Province of China to perform a fixed effect regression. It was found that economic growth target constraints and economic growth target gaps between countries and provinces, or between provinces and cities have a significant impact on the quality of public occupational health. The non-linear relationship between economic growth target setting and the quality of public occupational health was then discussed in detail, and the reliability of basic conclusions drawn was ensured by robustness and endogeneity tests. The results show that the effect of economic growth target constraints and gaps on the quality of public occupational health shows a “U-shaped” trend at both the provincial and city levels, which initially promotes and, eventually, inhibits. This relationship is closely related to the current economic system reforms, administrative reforms, and social transformation in China. Therefore, in emphasising high-quality economic development, the government should fully consider the actual state of the development of jurisdictions in setting economic goals to improve the quality of public occupational health in an orderly manner.

## Introduction

The purpose of this article is to analyse whether local economic growth target setting (measured by economic growth target constraints and gaps) has an impact on the quality of public occupational health (measured in reverse by the death rate of 100 million yuan of GDP of occupational safety accidents and the number of occupational safety accident deaths) at the provincial and city levels. Data released by the International Labour Organisation in 2000 show that between 1990 and 1999, the number of industrial and commercial deaths, and the mortality rate (except Russia) for industrialised countries and countries with the former European economies in transition decreased annually. The number of work-related deaths and death rates in China also decreased from 20,327 and 14.5 per 100,000 in 1990 to 14,660 and 9.4 per 100,000 in 1999. However, compared to that of developed countries, the state of industrial accidents in China is not optimistic. In 2007, there was still a large gap between China and developed countries in terms of the number of deaths caused by accidents. The death rate in China of 100 million yuan of GDP in occupational safety accidents is 10 times that of developed countries, and the accident death rate of the country of 100,000 people in the mining and commerce industry is more than two times that of developed countries. With this, it can be said that public occupational health management in China is at a relatively low level.

Economic growth is one of the targets of macroeconomic management. Since 1950, at least 49 economies, both developed and developing, have or had regularly announced growth targets, including China, India, and the European Union. Other countries have had such targets ratified by their parliaments. Since the reform and opening-up policy of China, competition among governments at all levels concerning economic growth targets has been an important driving force behind sustained and rapid economic growth (1). Every year, local governments of all levels adjust their economic growth targets according to the actual conditions of their respective areas to take into more comprehensive consideration the factors that may affect economic and social development and thus, flexibly set different targets at a certain stage, thereby forming a socialist GDP target management system with Chinese characteristics. Since the beginning of this century, improving the quality of public occupational health has become a top priority for local governments of all levels. Detailed policy plans and measures have been formulated in urban management, social governance, emergency management, and other fields. However, from the results of practise in China, the quality of public occupational health has not effectively improved.

The question of interest of this article concerns how local economic growth target setting affects the quality of public occupational health. Is the effect positive or negative? This article attempts to integrate economic growth target setting and occupational health management into an analytical framework and analyses the influencing factors of the quality of public occupational health from the perspective of economic growth target constraints and gaps between countries and provinces or between provinces and cities. Compared to existing research, the marginal contribution of this article covers two aspects. This article shows that occupational health problems in China from an economic growth target constraint and gap double perspective, which is not only beneficial for understanding occupational health management but also for making up the limitations of research on economic growth target management. In terms of research data and methods, economic growth target data from the government work reports of 31 provinces in China and 21 cities of Guangdong Province in China for 2001–2019 are manually collated. This article uses a fixed-effect model, the instrumental variable method, and the generalised method of moments combined with a variety of robustness testing methods to ensure the reliability of the basic conclusions drawn.

The structure of this article is as follows. Section Literature review reviews the existing literature, and Section Economic growth target setting and the quality of public occupational health carries on the theoretical analysis and proposes a hypothesis. Section Methodology introduces the empirical method applied. Section Data presents our empirical data and variables. Section Empirical results analyses our empirical results. Section Conclusions concludes.

## Literature Review

Economic target setting began in the 1950s when China was still a planned economy. After years of economic restructuring, China has abolished most of its past planning practises, but the 5-year plan and annual plan are still adopted through the “Outline of the Five-year Plan for National Economic” and “Social Development and Government Work Report.” As a guideline, such planning is no longer as mandatory and binding as it was during the planned economy era. For this reason, many scholars believe that non-binding economic target setting is redundant because it does not convey the intended message. In fact, government officials announce growth targets to provide a blueprint for economic development. When the Asian financial crisis hit in 1998, the central government set a growth target of 8% and asked local governments of all levels to pledge to meet it. At the same time, the World Bank, the International Monetary Fund, and other international financial institutions can make or adjust the forecasts for the Chinese economy or global economy according to the economic growth target announced by China (2).

Through the transition of China from a planned economy to a market economy, target setting has been an important management tool for the implementation of economic and public policies (3). Especially since the 1980s, target responsibility assessment has been gradually applied in China. The target is considered the “starting point” and “entry point” for higher-level government to manage lower-level government entities, is the main way for governments of all levels to allocate and supervise targets and tasks, and provides specific institutional guarantees for the realisation of various targets and tasks (4). The political economy of China has two very distinctive features. First, the promotion of local officials in China is closely related to the economic growth of their jurisdictions (5–7), which leads to fierce competition. Second, local officials play a leading role in promoting local economic development (8–11). In the Chinese economy, the guidance of the central government on national economic construction and development work is achieved through the formulation of economic growth target assessment tasks (12), and further through the encouragement and governance of governments of all levels along with their officials (6). Under the yardstick competition system, local officials in China engage in a “promotion race” in which they compete with their peers for faster economic growth because the central government will use relative performance evaluations to reward the best, or at least better, officials for promotion (3, 5, 6, 13). Under the mechanism of “promotion championship” with economic development as the main goal, the higher-level government shows a preference for economic growth by setting economic growth targets to encourage the lower-level government to improve the economic development of their jurisdictions by setting corresponding economic growth targets and to ensure that they succeed in the final relative performance assessments (2).

Local governments often need to make a trade-off between economic growth targets and the quality of economic development. When a government chooses to use a factor input policy to achieve an economic growth target, it will have to compromise the quality of economic development. As an important facet of high-quality economic development, the factors affecting the quality of occupational health have also attracted the attention of scholars. First, to explain the reason for low occupational health level from the perspective of enterprise management, Chen et al. (14) performed a statistical analysis of 410 major gas explosion accidents occurring from 1980 to 2000 and found that the proportion of accidents caused by human factors exceeded 96.59%, indicating that occupational health is a management problem rather than an unsolvable technical problem. Second, scholars have analysed the reasons for low occupational health levels from the perspective of economic policy. The inadequate protection of property rights is a key factor. Mine accidents in China mainly occur in small and medium-sized coal mines dominated by township coal mines, because they lack clear property rights, consistent standards, adequate safety inputs, and stable occupational health conditions (15). Third, scholars have sought to explain the reasons for low occupational health levels in terms of regulation systems. One view holds that a key reason for frequent safety accidents is a lack of strict supervision and that the establishment of coal regulatory agencies with vertical management can effectively reduce mine accidents (16). The other view is that, under the premise of emphasising GDP assessment and information asymmetry, local governments, for the benefit of political promotion and fiscal decentralisation, conspire with enterprises to encourage enterprises to choose low-cost and high-risk production methods to increase production, resulting in a decline in occupational health (17, 18). Another point of view is that the relevant governance policies are the key factors that affect the quality of occupational health. For example, “death indicators” encourage local governments to pay more attention to mine disaster management and significantly reduce the number of casualties (19). After the adoption of the “one vote no” policy, the overall work-related mortality rate of enterprises decreased by 50% (17), indicating that such a policy could indeed reduce the likelihood of the number of deaths exceeding the set limit, although the effect was only significant at the 10% level (20). Appropriate strong regulations can indeed improve occupational health quality (2), but excessively strict regulation policies cannot achieve the goals of protecting workers and improving safety levels (21). Despite the lack of a binding legal effect, soft regulation is considered to be an important governance tool for improving the quality of occupational health, especially when combined with strong regulation (22, 23).

According to existing studies, the economic growth target plays an important role in the economic field. On one hand, the significant impacts of fiscal decentralisation, official incentives, and other factors on the economic growth target have been fully verified. On the other hand, the significant impact of the economic growth target on productive indicators in the macroeconomic field has gradually attracted the attention of scholars, but attention to non-productive indicators is far from sufficient. Although relevant studies have noted the impact of economic growth targets on high-quality economic development, they have not focused on specific outcome indicators, such as the quality of public occupational health. Focusing on economic growth target constraints and gaps, this article analyses the impact of economic growth targets on non-productive fields based on provincial-level and city-level panel data and focuses on the special case of the quality of occupational health, providing a new research focus for economic growth target management.

## Economic Growth Target Setting and the Quality of Public Occupational Health

According to the target setting theory, target setting has a guiding and motivating effect on organisational and individual behaviours. It can encourage organisations and individuals to pay attention to performance output, improve task performance, and regularly track target tasks. Target setting encourages communication between superiors and superiors on the completion of tasks to promote the completion of target tasks (24). However, this form of target motivation is not always positive. Bevan and Hood (25) found a negative impact of target setting in the health field, which may lead hospitals to use any means to achieve their goals, and the authors refer to this phenomenon as output distortion. Kelman and Friedman (26) argue that there is a government effort to neglect the dimensions of performance that are not easily measured and to make the measurable dimensions better. According to the theory of multiple assessment objectives, when the subject is faced with multiple assessment objectives, priority resources are often invested into those indicators with greater assessment weight and higher performance (27). However, the effects of the transition of local governments and the limited tenure of officials require resource investment to be quickly transformed into target tasks.

Holmstrom and Milgrom (28) analysed a situation in which an agent is engaged in multiple tasks. The authors argue that when faced with multiple tasks, agents should not only allocate their motivation to different tasks but, more importantly, should also allocate attention to different tasks. Therefore, when facing the two goals of economic growth and security governance, local governments must first make a choice to focus more on one goal and then determine their degrees of efforts, which are, of course, determined by the external constraints of local governments in place at the time. In addition, Holmstrom and Milgrom (28) further indicate that the difficulty of measuring the effort required to complete a task can have a significant impact on ordering. Chinese scholars Li and Zhou (6) put forward a more specific point of view in arguing that Chinese local governments are faced with multiple goals when they are in power, which is mainly reflected by the fact that local officials under promotion incentives only pay attention to indicators that can be assessed and pay no attention to indicators outside the assessment scope or to the consequences that are difficult to measure. We believe that local governments have also followed these rules when coordinating the objectives of economic growth and occupational health management.

Local governments in China face a multitasking problem in setting and achieving economic growth targets for their jurisdictions. Nie et al. (29) found the number of mining accidents and the number of deaths during two sessions to be relatively low. The reason is that the main target of local governments during two sessions is to maintain stability, causing the number of mining accidents and the number of deaths to decrease. This has led to an increase in the number of mining accidents and deaths. Therefore, in terms of economic efficiency, promoting economic growth and improving the quality of public occupational health are considered to be in conflict with each other (18). Because the system or policy adopted affects the incentive structure, and as the incentive structure will affect behaviour (30), enterprises tend to ignore the quality of public occupational health, so that the local government can create more economic growth and fiscal revenue to meet the economic growth target, but this can often lead to frequent enterprise safety accidents. Especially under the constraint of economic growth targets, local governments are motivated to pursue the maximisation of financial revenue and investment benefit and invest limited resources in the fields that can bring economic growth effects in the short term, while the non-productive fields, such as occupational health management, have difficulty entering the objective performance functions of government departments. When the limited allocation and sequencing of resources are required among multiple objectives, productive areas are often prioritised over unproductive areas (31), resulting in insufficient attention to public occupational health management. The above analysis shows that an economic growth target will lead to an overemphasis of the local government on economic indicators, while the indicators related to occupational health management will not receive enough attention, which is not conducive to the improvement of safety conditions. That is, the strengthening of economic growth target constraints reduces the quality of public occupational health.

However, the effect of economic growth objectives on the quality of public occupational health may not be linear. With the introduction of a series of reform systems in developing countries, the traditional development mode has difficulty providing sustained impetus for economic growth, causing the performance objective function of government to change. Especially when high-quality economic development gradually attracts more attention, the achievement of higher-quality, more efficient, more equitable, and more sustainable economic development has become a problem for most developing country government departments. China is a developing country, and economic development is still its main focus, especially under the constraints of economic growth targets. Economic growth efficiency is related to the political interests of officials. However, safety accidents are the bottom-line requirement, which is also closely related to the political careers of officials (17). It is very important for local governments to deal with the relationship between economic growth and occupational health management in the process of economic growth target adjustment. Under the constraints of economic growth, in order to improve economic performance and avoid safety incidents, local governments closed down enterprises with low economic efficiency and high accident rates (29), and increased the weight of occupational health management in government, and increased the weight of occupational health management in government (32). Under the new target assessment system, the governance performance of local officials in the field of occupational health constitutes an important political promotion incentive and is conducive to improving the quality of occupational health (33).

Therefore, it is an important goal for local governments to seek a more efficient economic development mode and reduce safety accidents that occur with economic growth. Especially in recent years, technological, management, and system innovation have been important driving forces of economic growth (34). “Competition for innovation” has thus begun to appear (35), which is undoubtedly beneficial for the improvement of public occupational health. By promoting cross-regional cooperation, local governments cannot only promote the coordinated development of regional economies and cross-regional cooperative governance among governments (36) but also improve the efficiency of safety supervision (37–39). Therefore, when the economic growth target is at a high level, the strengthening of the economic growth target constraint is conducive to the improvement of public occupational health. From the above analysis, under limited economic growth target constraints, the productivity index more easily affects the local government performance target function, and “compliance costs” maximises the economic benefit of local governments to encourage enterprises to select the low cost and high-risk production mode to increase production, reducing the quality of the occupational health (18). When the economic growth target constraint level is higher, occupational health management serves as a productivity index and public product that is gradually recognised from the objective functions of local government performance. In addition, the improvement of the economic growth target reverses the transmission government department for technical innovation, cross-regional cooperation, and management to achieve economic growth target tasks and occupational health management. Therefore, the relationship between economic growth objective constraints and the quality of public occupational health may be non-linear. When the economic growth target constraints are at a low level, it has a negative impact on the public quality of occupational health. When the economic growth target constraints are at a high level, it has a positive impact on the public quality of occupational health. On this basis, the following hypothesis is proposed:

Hypothesis 1: The effect of local economic growth target constraints on the quality of public occupational health is non-linear.

Due to the differences in the resource endowments, development stages, and economic environments of different regions, it is objectively necessary to set and adjust the economic growth target according to actual local conditions. However, under the promotion championship system and relative performance appraisal system, when the government sets an economic growth target, it often anchors target setting by higher or other statistical government departments because the macroeconomic policies and resource allocation authority of lower-level governments are guided and controlled by higher-level governments. When formulating economic growth targets, lower-level governments are bound to have strategic responses in guiding the policies of higher-level governments. A cadre evaluation system based on relative economic performance has led to local government leaders competing with each other on economic targets. Higher levels of government use targets to convey their preference for economic growth among a range of responsibilities (2). Targets setting by higher government departments can enable lower government departments to improve target setting without complete information. To stand out from the competition, lower-level government leaders often use their position relative to the economic growth targets set by the higher-level government as a measure of how much effort they are dedicating to the competition (3). As a result, career-oriented local leaders have an incentive to set higher targets than their superiors and work harder to achieve those targets. When GDP evaluation is given priority to, under the “tournament” promotion mechanism of region economy growth due to local officials to “hooking” the promoted height, local officials set high economic growth targets for the higher-level government with the release of the “ability to signal” (6). Li et al. (2) investigated the strategy of target setting from the perspective of government hierarchy relationships of economic growth and found that all levels of government show a gap in economic growth targets. As a result, growth targets cause the consequences such as redundant construction, investment overheating, and environmental pollution (3), frequent safety accidents (40), and other governance difficulties, affecting the high-quality development of the local economy. Similarly, the relationship between the economic growth target gap and the quality of public occupational health may be non-linear. When the gap is small, an increase in the gap deteriorates the quality of public occupational health. When the gap is large, an increase in the gap improves the quality of public occupational health. On this basis, the following hypothesis is proposed:

Hypothesis 2: The effect of the economic growth target gap on the quality of public occupational health is non-linear.

## Methodology

To test the relationship between the economic growth target setting and the quality of public occupational health, we applied the following time and regional fixed-effect model:


(1) $$\begin{array}{r} Y_{it}=\theta_{0}+\theta_{1}target_{it}+\theta_{2}target_{it}^{2}+\underset{it}{\sum }\;\theta_{it}Control\\ \;+\mu_{i}+\delta_{i}+\varepsilon_{it} \end{array}$$

(2) $$\begin{array}{r} Y_{it}=\theta_{0}+\theta_{1}{target\text{\_}h}_{it}+\theta_{2}{target\text{\_}h}_{it}^{2}\\ \;+\underset{it}{\sum }\;\theta_{it}Control+\mu_{i}+\delta_{t}+\varepsilon_{it} \end{array}$$

where *i* represents the province (city) and *t* represents the year. *Y*_*it*_ represents the quality of public occupational health, *target*_*it*_ represents the economic growth target constraint, and *target*_*h*_*it*_ represents the economic growth target gap. $target_{it}^{2}$ and ${target\text{\_}h}_{it}^{2}$ represent the square terms of the economic growth target constraint and gap, respectively. *Control* represents some other control variables, including the level of economic development (ln _*gdp*), industrial structure (*Industrial*), fiscal decentralisation (*Fiscal*), fixed asset investment (*Invest*), and transition effect (*Change*). μ_*i*_ represents the unobserved factors that do not change with time in each province (city) to control the regional fixed effect. δ_*t*_ controls the fixed time effect. ε_*it*_ is the random perturbation term.

## Data

This article analyses the relationship between economic growth target setting and the quality of public occupational health at the provincial and city levels. For the growth target data, we collated 589 government work reports from 31 provincial governments of China, and 399 government work reports from 21 city governments of Guangdong Province in China between 2001 and 2019. We use the sampling data of 21 cities in Guangdong Province to consider two aspects. The first is data availability since most of the studied cities did not release occupational health management-related indicators, but the “Guangdong Statistical Yearbook” and the “Statistical Bulletin of National Economic and Social Development” of the 21 cities released the related indicators. Secondly, we considered the representativeness of our sample. From the start of the new century, occupational safety accidents occurring in transportation and warehousing, trade and manufacturing, construction and other fields, and the economic and social development levels of the Pearl River Delta and the eastern, western, and northern of Guangdong Province are varied, reflecting patterns found across China. Government work report data were mainly derived from the official websites of provincial and city governments, with some data taken from provincial and city statistical yearbooks, and other data drawn from the “China Statistical Yearbook” “Guangdong Statistical Yearbook” and the “Statistical Bulletin of National Economic and Social Development” content of the studied provinces and cities.

### Dependent Variables

In 2004, the Work Safety Commission of the State Council of China listed the death toll from production safety accidents in industrial, mining, and trade enterprises. The death toll from coal accidents and the death rate of 1 million tonnes of coal mines as the assessment indicators for local occupational health management. The commission also used the death rate of 100 million yuan of GDP, one hundred thousand people, and one hundred thousand workers in industrial, mining, and trade as the record indicators. Based on principles of science and availability, this article uses a death rate of 100 million yuan of GDP for occupational safety accidents (death rate, *Ln*_*rate*), and the death toll of occupational safety accidents (number of deaths, *Ln*_*death*) to reverse estimate the quality of public occupational health from the absolute and relative numbers.

### Independent Variables

The first main independent variable considered is the economic growth target constrain. Government work reports reflect the official policy program of local governments and good authority. Under normal circumstances, the provinces (autonomous regions and municipalities) and cities of the National People's Congress meet in the middle of each year in late January and February. The meetings involve reviewing the main achievements of the previous year, announcing various targets for the upcoming year, determining the target for economic growth and the growth of general public budget revenue, and formulating detailed supporting policies for these targets. At the same time, the report produced on the work of the government will serve as the basis for the future assessment of all government departments. We use the values of annual economic growth targets published in government work reports to measure economic growth target constraints.

The second main independent variable is the economic growth target gap. In this article, the economic growth target gap covers two aspects. The first is the economic growth target gap between the country and a province, which is measured as the difference between a provincial economic growth target and the national economic growth target. The other is the economic growth target gap between a province and a city, which is measured as the difference between the economic growth target of a city and that of the corresponding province. Based on data from the 2001–2019 government work reports, it is estimated that the provincial economic growth target is on average ~2% points higher than the national economic growth target for each year, while the provincial economic growth target is on average ~3% points higher than the provincial economic growth target for each year.

From the provincial and city-level government work reports, the text of the economic growth target is more complicated to express. This article deals with the special expression of the economic growth target. For example, if the economic growth target is a clear number without interval properties, such as “left and right,” “above” or “guarantee,” we take this value as an indicator to measure the economic growth target. For an economic growth target expressed in a range, such as “7–7.5%,” “10% forecasted growth and goal of 11%,” and so on, we take the average or the lower limit as an indicator to measure the economic growth target.

### Regional Characteristic Control Variables

Considering the large differences in economic and social characteristics found among the different provinces or cities, other control variables are added to more accurately analyse the impact of economic growth target setting on the quality of public occupational health. The first is the level of economic development. Low input inadequacy and production technology levels result in frequent safety accidents (41, 42), and stronger economic development, and improved safety management inputs and technical levels. We control the impact of the economic development level on the quality of public occupational health by adding GDP. The second variable considered is industrial structure. Safety accidents have obvious industrial characteristics. Both the mining industry and the construction industry belong to industries with poor safety levels, and the fatality rate of the mining industry is much higher than the social average. In this article, the ratio of the total output value of the tertiary industry to the total output value of the secondary industry is used to measure the industrial structure (43). The third variable considered is the level of fiscal decentralisation. The Chinese fiscal decentralisation system entrusts local governments with a certain degree of revenue autonomy, which leads to the preference of local governments for productive expenditures which is not conducive to the investment in security governance (6, 10). In this article, the fiscal autonomy index is used to measure the degree of fiscal decentralisation, which is calculated by dividing budgetary revenue by budgetary expenditure. The fourth variable considered is fixed asset investment. Improving fixed asset investment can help enterprises upgrade equipment and pursue technical transformation to improve the quality of public occupational health and reduce the number of safety accidents and deaths (44). This article uses the proportion of fixed assets investment of regional GDP as a measure. The fifth variable considered is the transition effect. Under the cadre appointment system, officials at all levels in China transition in relatively fixed years (29), so the 4 years of transition of 2002, 2007, 2012, and 2017 are further controlled to investigate the influence of political cycles on the number of safety accidents. To ensure the accuracy of the measurement results, the quality of public occupational health and economic development levels were logarithmically processed. Descriptive statistics of the related variables are shown in Table 1.

**Table 1:** Descriptive statistics of the variables.

**Variable**	**Max**		**Min**		**Mean**		**Std. Dev**.	
	**Provincial level**	**City level**	**Provincial level**	**City level**	**Provincial level**	**City level**	**Provincial level**	**City level**
Ln_rate	1.277	1.163	−5.657	−4.510	−1.384	−1.488	1.303	1.203
Ln_death	10.139	7.771	4.083	3.605	7.667	5.590	0.913	0.759
Target	15.000	24.000	4.500	5.500	9.443	11.117	1.785	3.000
Target^2^	225.000	576.000	20.250	30.250	92.355	132.567	34.716	73.651
Target_h	7.000	15.000	−1.500	−2.000	2.127	2.722	1.453	2.526
Target_h^2^	49.000	225.000	0.000	0.000	6.634	13.775	8.036	22.870
Ln_gdp	11.626	10.270	4.984	4.578	9.042	7.064	1.229	1.175
Industrial	5.168	2.626	0.494	0.431	1.045	0.932	0.559	0.327
Fiscal	0.951	1.035	0.053	0.135	0.496	0.542	0.202	0.235
Invest	147.116	128.862	20.005	11.332	64.359	43.726	25.884	21.203
Change	1.000	1.000	0.000	0.000	0.211	0.211	0.202	0.408

## Empirical Results

### Basic Analysis Results

**Figure 1:**
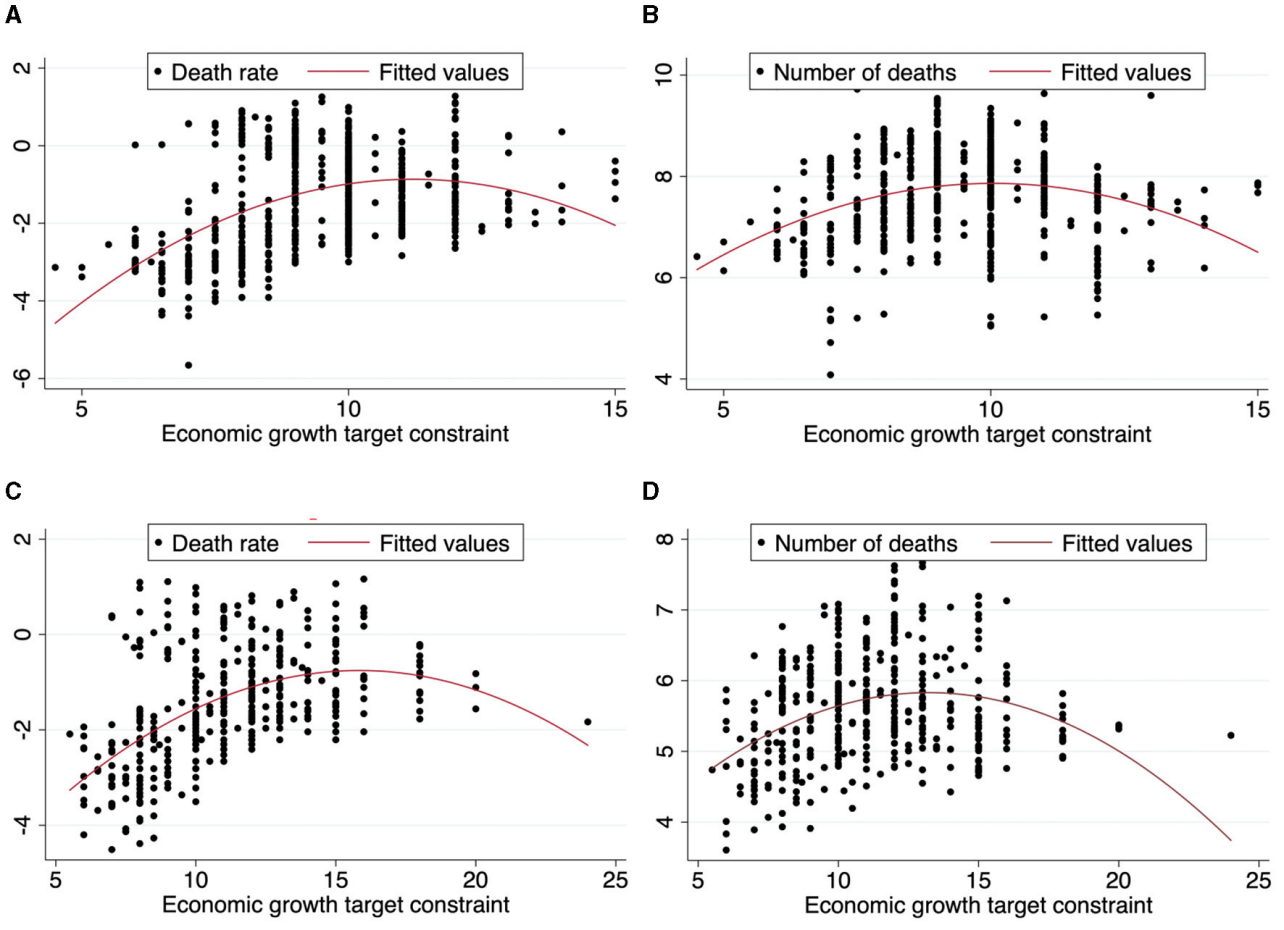
The scatter diagram of the relationship between economic growth target constraint and the quality of public occupational health. **(A)** Economic growth target constraint and death rate at provincial level. **(B)** Economic growth target constraint and number of deaths at provincial level. **(C)** Economic growth target constraint and death rate at city level. **(D)** Economic growth target constraint and number of deaths at city level.

To directly observe the correlation between economic growth target setting and the quality of public occupational health, we conducted a scatter diagram analysis of the economic growth target constraint with the death rate and the number of deaths (as shown in Figure 1). Figures 1A,C show an “inverted U-shaped” correlation between the economic growth target constraint and death rate at the provincial and city levels, respectively. Figures 1B,D also show an “inverted U-shaped” correlation between the economic growth target constraint and the number of deaths at the provincial and city levels, respectively. All results indicate a “U-shaped” correlation between the economic growth target constraint and the quality of public occupational health, which preliminarily verifies Hypothesis 1 of this article reporting a “U-shaped” non-linear relationship between the economic growth target constraint and the quality of public occupational health.

**Figure 2:**
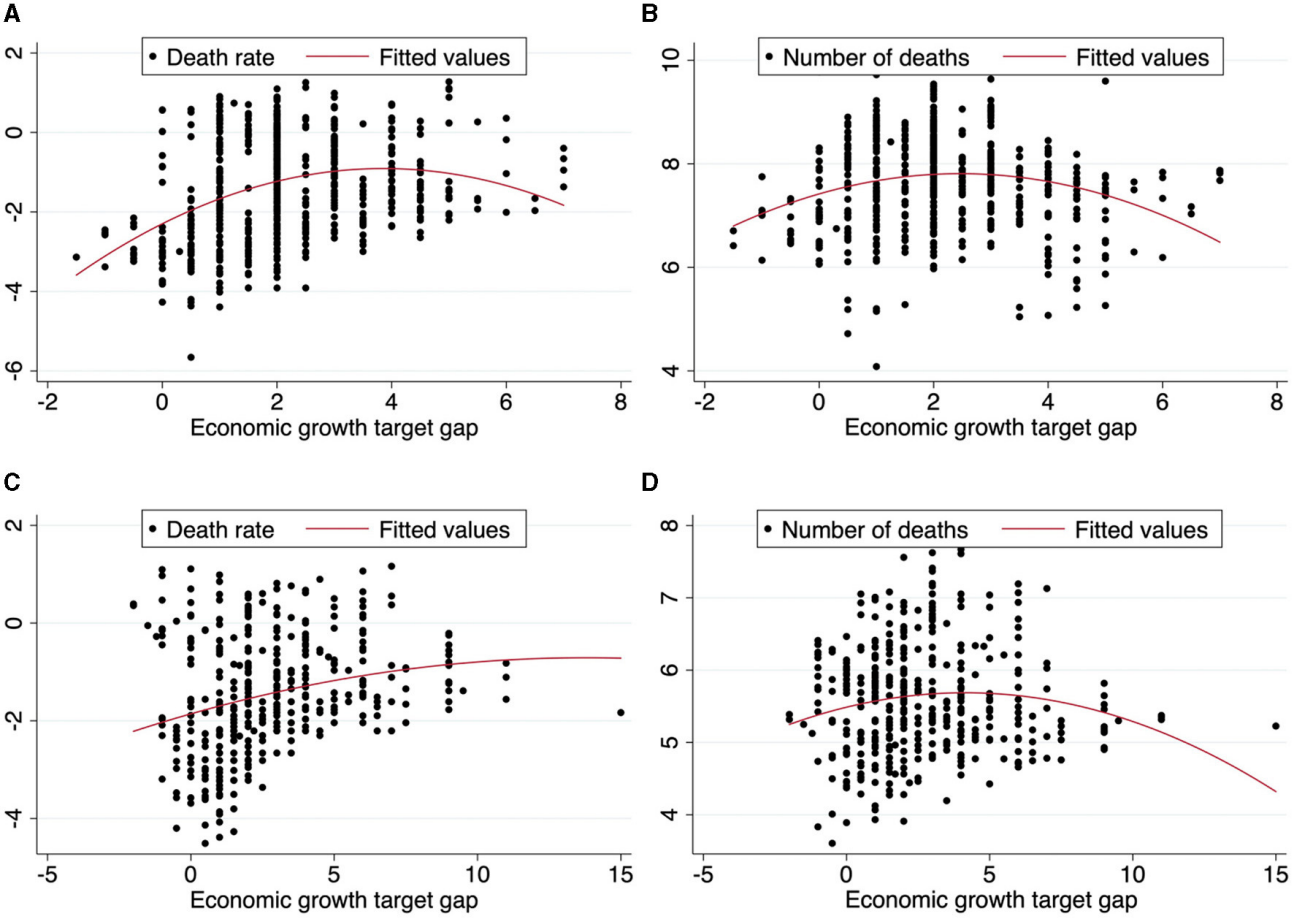
The scatter diagram of the relationship between economic growth target gap and the quality of public occupational health. **(A)** Economic growth target gap and death rate at provincial level. **(B)** Economic growth target gap and number of deaths at provincial level. **(C)** Economic growth target gap and death rate at city level. **(D)** Economic growth target gap and number of deaths at city level.

A scatter diagram analysis of the economic growth target gap, accident rate, and the number of deaths is also carried out (as shown in Figure 2). From Figures 2A,C, there is an “inverted U-shaped” correlation between the economic growth target gap and death rate, and Figures 2B,D also show an “inverted U-shaped” correlation between the economic growth target gap and number of deaths, which indicates a “U-shaped” correlation between the economic growth target gap and the quality of public occupational health. In addition, the correlation at the provincial and city levels shows the same trend. Thus, Hypothesis 2 of this article is preliminarily verified: there is a “U-shaped” non-linear relationship between the economic growth target gap and the quality of public occupational health.

### Baseline Regression Results

Table 2 reports the baseline regression results for the impact of local economic growth target setting on the quality of public occupational health. Columns (1, 2) and (5, 6) show the effects of economic growth target constraint at the provincial and city levels, respectively. Columns (3, 4) and Columns (7, 8) show the effects of the economic growth target gap at the provincial and city levels, respectively. At the provincial and city levels, when the accident rate and the number of deaths are set as dependent variables, the regression results show that the coefficient of the economic growth target constraint is positive, and the square of the coefficient of the economic growth target constraint is negative. All models have at least a 5% significance level, indicating that the economic growth target constraint and the quality of the occupational health have a “U-shaped” non-linear relationship. The coefficient of the economic growth target gap is positive, and the coefficient of the square of the economic growth target gap is negative. All models passed the significance level of at least 10%, indicating that the influence of the economic growth target gap on the quality of public occupational health also shows a “U-shaped” non-linear relationship. From the results of the control variable, economic development, industrial structure, fiscal decentralisation, fixed asset investment, and the transition effect affect the quality of occupational health to varying degrees, and differences are also shown for the samples of the provincial and city levels.

**Table 2:** The result of panel regression.

	**Provincial level**				**City level**			
	**Ln_rate**	**Ln_death**	**Ln_rate**	**Ln_death**	**Ln_rate**	**Ln_death**	**Ln_rate**	**Ln_death**
	**(1)**	**(2)**	**(3)**	**(4)**	**(5)**	**(6)**	**(7)**	**(8)**
Target	0.241**	0.271***			0.064**	0.071**		
	(0.099)	(0.103)			(0.028)	(0.028)		
Target^2^	−0.015***	−0.017***			−0.002**	−0.002**		
	(0.005)	(0.005)			(0.001)	(0.001)		
Target_h			0.073*	0.083**			0.028**	0.039***
			(0.039)	(0.040)			(0.011)	(0.011)
Target_h^2^			−0.027***	−0.028***			−0.004***	−0.004***
			(0.006)	(0.006)			(0.001)	(0.001)
Ln_gdp	−0.888***	0.157	−0.869***	0.183	−0.703***	0.209*	−1.441***	−0.472***
	(0.256)	(0.265)	(0.250)	(0.259)	(0.126)	(0.126)	(0.023)	(0.023)
Industrial	−0.011	−0.094	0.004	−0.080	−0.128*	−0.131*	−0.308***	−0.316***
	(0.075)	(0.078)	(0.074)	(0.077)	(0.067)	(0.067)	(0.069)	(0.069)
Fiscal	0.960**	1.577***	0.947**	1.566***	−0.716***	−0.721***	0.084	0.110
	(0.430)	(0.446)	(0.426)	(0.443)	(0.226)	(0.226)	(0.175)	(0.175)
Invest	−0.003**	−0.003*	−0.003**	−0.003*	−0.006***	−0.005***	−0.007***	−0.006***
	(0.001)	(0.001)	(0.001)	(0.001)	(0.001)	(0.001)	(0.001)	(0.001)
Change	−2.648***	−0.950***	−2.649***	−0.959***	−1.613***	−1.470***	−0.045*	−0.047*
	(0.174)	(0.181)	(0.165)	(0.171)	(0.268)	(0.268)	(0.027)	(0.027)
Region FE	yes	yes	yes	yes	yes	yes	yes	yes
Year FE	yes	yes	yes	yes	yes	yes	yes	yes
Constant	5.976***	5.206***	6.731***	6.044***	4.662***	5.116***	9.202***	9.383***
	(1.852)	(1.924)	(1.904)	(1.979)	(0.790)	(0.791)	(0.169)	(0.169)
R–squared	0.916	0.609	0.917	0.615	0.973	0.859	0.963	0.804
N	589	589	589	589	399	399	399	399

***, **, and * respectively indicates significance at the 1, 5 and 10% level. Standard error in parentheses.

The above results show that when the economic growth target constraint and gap are less pronounced, the productive indicator can enter the objective function of the local government. Coupled with the existence of “compliance costs,” the strengthening of economic growth target constraints and gaps prompted a local government to “crowd out” occupational health management, relax safety regulations, and neglect high-risk production behaviours of enterprises to obtain greater economic benefits, leading to frequent safety accidents. When the level of economic growth target constraint and gap are significant, the strengthening of the economic growth target constraint and gap forces local governments to innovate the economic development mode and complete the growth target task through technological innovation, industrial structure upgrading, transregional cooperation, and governance, improving the quality of occupational health.

### Endogeneity Tests

Although we alleviate the problem of missing variables to some extent by adding bidirectional fixed effects to the baseline regression results, the endogeneity problem cannot be solved when the quality of occupational health has an inverse relationship with the economic growth target setting. To alleviate the endogeneity problems caused by missing variables or reverse causality, we adopt the instrumental variable method and generalised method of moments. In our endogeneity test, we construct different instrumental variables for economic growth target constraints and gaps of different levels. In this article, the lag period of the economic growth target constraint and gap is taken as the economic growth target constraint and gap instrumental variable (IV1 and IV2, IV3 and IV4) at both the provincial and city levels (45). The realisation of the economic growth target in the last period will affect the financial revenue of local governments, foreign direct investment, and fixed asset investment and thus, affect the quality of public occupational health.

The results of the endogeneity test (as shown in Table 3) show that the Durbin-Wu-Hausman test results reject the null hypothesis of no endogeneity problem at the significance level of 5%, indicating endogeneity problems between the economic growth target constraint and gap and the quality of occupational health in the econometric model. A two-stage least squares estimate (2SLS) test also shows that the local economic growth target constraint and gap and quality of public occupational health show a “U-shaped” non-linear relationship. Considering the nature of the endogenous problem, our conclusions are still valid. To ensure the rationality of the selection of instrumental variables, an underidentification test is conducted for all models, and all models reject the null hypothesis with an underidentification problem of 1%. Second, the weak instrumental variable test shows that the Cragg-Donald Wald F statistic of the model is significantly larger than the critical value of 16.39 of the F value at the 10% bias level (46), indicating that there is no weak instrumental variable problem. The above results indicate that the instrumental variables used in this article are valid.

**Table 3:** Regression results of the instrumental variable method.

	**Provincial level**				**City level**			
	**IV1**		**IV2**		**IV3**		**IV4**	
	**Ln_rate**	**Ln_death**	**Ln_rate**	**Ln_death**	**Ln_rate**	**Ln_death**	**Ln_rate**	**Ln_death**
	**(1)**	**(2)**	**(3)**	**(4)**	**(5)**	**(6)**	**(7)**	**(8)**
Target	0.682***	0.789***			0.190***	0.199***		
	(0.173)	(0.179)			(0.072)	(0.072)		
Target^2^	−0.038***	−0.042***			−0.007***	−0.007***		
	(0.008)	(0.008)			(0.003)	(0.003)		
Target_h			0.279***	0.325***			0.066***	0.073***
			(0.080)	(0.083)			(0.025)	(0.025)
Target_h^2^			−0.069***	−0.074***			−0.007***	−0.008***
			(0.012)	(0.012)			(0.003)	(0.003)
Control variable	yes	yes	yes	yes	yes	yes	yes	yes
Region FE	yes	yes	yes	yes	yes	yes	yes	yes
Year FE	yes	yes	yes	yes	yes	yes	yes	yes
Constant	2.250	3.849	4.946*	6.871**	1.880	2.429*	2.474**	3.053**
	(2.406)	(2.494)	(2.698)	(2.799)	(1.349)	(1.350)	(1.232)	(1.233)
Durbin-Wu-Hausman	13.477	7.919	7.827	15.336	8.856	7.472	54.493	25.910
(*P*-value)	(0.001)	(0.019)	(0.020)	(0.001)	(0.012)	(0.024)	(0.000)	(0.000)
LM statistic	147.356		131.721		54.503		62.916	
(*P*-value)	0.000		0.000		0.000		0.000	
Wald F statistic	97.812		83.975		30.089		35.728	
R^2^	0.907	0.596	0.905	0.587	0.969	0.846	0.970	0.848
N	558	558	558	558	378	378	378	378

***, ** and * respectively indicates significance at the 1, 5, and 10% level. Standard error in parentheses.

Most of the variables of economic life have an “inertia” effect, and the quality of public occupational health in the current period may affect that in the next period. Therefore, it is necessary to add the lag term of the dependent variable to investigate the dynamic effect of the quality of public occupational health. However, due to the endogeneity problem caused by the lag term of the dependent variable included in the independent variable, we further adopt the system generalised method of moments (SYS-GMM) and difference generalised method of moments Model (DIFF-GMM) for estimation. The results are shown in Table 4. The *Sargan* statistic of the overrecognition test is not significant, indicating that the selection of instrumental variables is valid, and AR (2) cannot reject the null hypothesis at the level of 5%, which means that the disturbance term has no autocorrelation, and the model setting and estimation method are reasonable (47). The lag term coefficients of each model are significantly positive, indicating that modelling with the lag term of the dependent variable added into the explanatory variable is still effective. The estimated results listed in Table 4 show that the coefficients of the economic growth target constraint and gap are positive, while the quadratic coefficients remain significantly negative, indicating that the benchmark regression results are still valid.

**Table 4:** Regression results of generalised method of moments.

	**Provincial level**				**City level**			
	***sys-gmm***	***diff-gmm***	***sys-gmm***	***diff-gmm***	***sys-gmm***	***diff-gmm***	***sys-gmm***	***diff-gmm***
	**(1)**	**(2)**	**(3)**	**(4)**	**(5)**	**(6)**	**(7)**	**(8)**
Lag.	0.914***	1.001***	0.890***	0.759***	0.661***	0.469***	0.687***	0.547***
	(0.021)	(0.040)	(0.015)	(0.019)	(0.049)	(0.062)	(0.047)	(0.059)
Target	0.160***	0.174***			0.050**	0.065***		
	(0.046)	(0.058)			(0.020)	(0.025)		
Target^2^	−0.008***	−0.007***			−0.003***	−0.003***		
	(0.002)	(0.003)			(0.001)	(0.001)		
Target_h			0.014*	0.054***			0.014*	0.001
			(0.008)	(0.016)			(0.008)	(0.008)
Target_h^2^			−0.004***	−0.005**			−0.001*	−0.002*
			(0.001)	(0.003)			(0.001)	(0.001)
Control variables	yes	yes	yes	yes	yes	yes	yes	yes
Constant	0.017	1.653	1.771***	9.207***	2.462***	3.910***	2.517***	3.888***
	(0.432)	(1.442)	(0.417)	(0.590)	(0.517)	(0.561)	(0.494)	(0.729)
AR(1)	0.041	0.043	0.042	0.040	0.003	0.002	0.003	0.003
AR(2)	0.100	0.094	0.103	0.101	0.547	0.493	0.568	0.536
Sargan Test	0.832	0.050	0.737	1.000	1.000	0.278	1.000	0.960
N	558	527	558	527	378	357	378	357

***, **, and * respectively indicates significance at the 1, 5, and 10% level. Standard error in parentheses; Only regression results with accident mortality as the dependent variable are reported here.

### Robustness Tests

In addition to using the instrumental variable method and generalised method of moments to address the endogeneity problem of the model, to further ensure the reliability of the benchmark regression results, this article will also carry out three aspects of robustness tests.

We reexamine the economic growth target constraint and gap to obtain Table 5. Specifically, if “expected growth of more than 10%” is cited in the text, the economic growth target is treated as 10.05%. If “expected 10% growth with a goal of 11%” appears in the text, we treat the economic growth target as 10.5% (as shown in Table 5).

**Table 5:** Robustness tests: change measurement method of economic growth target setting.

	**Provincial level**				**City level**			
	**Ln_rate**	**Ln_death**	**Ln_rate**	**Ln_death**	**Ln_rate**	**Ln_death**	**Ln_rate**	**Ln_death**
	**(1)**	**(2)**	**(3)**	**(4)**	**(5)**	**(6)**	**(7)**	**(8)**
Target	0.260***	0.294***			0.064**	0.071**		
	(0.098)	(0.102)			(0.028)	(0.028)		
Target^2^	−0.016***	−0.018***			−0.002**	−0.002**		
	(0.005)	(0.005)			(0.001)	(0.001)		
Target_h			0.076**	0.087**			0.027*	0.032**
			(0.039)	(0.040)			(0.014)	(0.014)
Target_h^2^			−0.027***	−0.029***			−0.002*	−0.002*
			(0.006)	(0.006)			(0.001)	(0.001)
Control variables	yes	yes	yes	yes	yes	yes	yes	yes
Region FE	yes	yes	yes	yes	yes	yes	yes	yes
Year FE	yes	yes	yes	yes	yes	yes	yes	yes
Constant	5.900***	5.139***	6.689***	6.018***	4.663***	5.118***	5.090***	5.588***
	(1.859)	(1.930)	(1.918)	(1.992)	(0.790)	(0.791)	(0.742)	(0.743)
R^2^	0.916	0.611	0.918	0.617	0.973	0.859	0.973	0.858
N	589	589	589	589	399	399	399	399

***, **, and * respectively indicates significance at the 1, 5, and 10% level. Standard error in parentheses.

We control the individual characteristics of officials to obtain Table 6. Related studies have found that different individual characteristics of officials represent differentiated promotion incentives, which have an impact on the quality of public occupational health (18, 29). This article considers the improvement of the quality of public occupational health as an achievement of both party committee leaders and administrative leaders. This increases the number of observations relative to the previous observations. We collected the individual characteristics of the party committee and administrative leaders, including their age, education, and work experience characteristics (city, provincial, and central government departments and enterprises). These data were mainly drawn from the “Party and Government Leading Cadre Database” and “Chinese Politicians Database,” and then we controlled the characteristics of officials for the impact of individual characteristics on the quality of public occupational health (as shown in Table 6).

**Table 6:** Robustness cheque: control the individual characteristics of officials.

	**Provincial level**				**City level**			
	**Ln_rate**	**Ln_death**	**Ln_rate**	**Ln_death**	**Ln_rate**	**Ln_death**	**Ln_rate**	**Ln_death**
	**(1)**	**(2)**	**(3)**	**(4)**	**(5)**	**(6)**	**(7)**	**(8)**
Target	0.227***	0.259***			0.058***	0.065***		
	(0.069)	(0.072)			(0.020)	(0.020)		
Target^2^	−0.014***	−0.016***			−0.002***	−0.002***		
	(0.003)	(0.003)			(0.001)	(0.001)		
Target_h			0.073***	0.085***			0.026***	0.031***
			(0.027)	(0.028)			(0.008)	(0.008)
Target_h^2^			−0.026***	−0.028***			−0.002***	−0.003***
			(0.004)	(0.004)			(0.001)	(0.001)
Control variables	yes	yes	yes	yes	yes	yes	yes	yes
Region FE	yes	yes	yes	yes	yes	yes	yes	yes
Year FE	yes	yes	yes	yes	yes	yes	yes	yes
Constant	5.822***	5.108***	6.601***	5.987***	4.341***	4.848***	4.618***	5.169***
	(1.307)	(1.357)	(1.344)	(1.395)	(0.582)	(0.584)	(0.554)	(0.556)
R^2^	0.918	0.620	0.920	0.626	0.974	0.861	0.974	0.862
N	1178	1178	1178	1178	798	798	798	798

*** indicates significance at the 1% level. Standard error in parentheses.

We control the effect of differences in administrative level to obtain Table 7. All provinces and cities in China have differences in administrative levels, which will specifically affect the local economy, society, and politics. We eliminated data from four municipalities in China, including Beijing, Tianjin, Shanghai, and Chongqing. At the city level, we eliminated two sub-provincial city samples, including Guangzhou and Shenzhen. The remaining samples were used for regression analysis to verify the reliability of the conclusion (as shown in Table 7).

**Table 7:** Robustness cheque: control the effect of differences at the administrative level.

	**Provincial level**				**City level**			
	**Ln_rate**	**Ln_death**	**Ln_rate**	**Ln_death**	**Ln_rate**	**Ln_death**	**Ln_rate**	**Ln_death**
	**(1)**	**(2)**	**(3)**	**(4)**	**(5)**	**(6)**	**(7)**	**(8)**
Target	0.280**	0.287**			0.066**	0.073**		
	(0.123)	(0.122)			(0.029)	(0.029)		
Target^2^	−0.018***	−0.018***			−0.002*	−0.002**		
	(0.006)	(0.006)			(0.001)	(0.001)		
Target_h			0.083*	0.090*			0.033***	0.038***
			(0.047)	(0.047)			(0.012)	(0.012)
Target_h^2^			−0.032***	−0.032***			−0.002**	−0.003**
			(0.007)	(0.007)			(0.001)	(0.001)
Control variables	yes	yes	yes	yes	yes	yes	yes	yes
Region FE	yes	yes	yes	yes	yes	yes	yes	yes
Year FE	yes	yes	yes	yes	yes	yes	yes	yes
Constant	−2.433	−2.440	−1.631	−1.587	3.655***	4.161***	3.989***	4.541***
	(1.695)	(1.688)	(1.689)	(1.682)	(0.806)	(0.811)	(0.759)	(0.763)
R^2^	0.908	0.626	0.910	0.633	0.973	0.854	0.973	0.855
N	513	513	513	513	361	361	361	361

***, **, and * respectively indicates significance at the 1, 5, and 10% level. Standard error in parentheses.

The above results show that no matter what robustness test method is adopted, the regression results are basically consistent with the abovementioned conclusions.

## Conclusions

This article examines the effect of local economic growth targets on the quality of public occupational health. Our panel fixed regression results show that the economic growth target constraint on the quality of public occupational health shows a U-shaped trend, first increasing and then decreasing. When the economic growth target constraint is at a low level, the economic growth target constraint has a negative impact on the public quality of occupational health. When the economic growth target constraint is at a high level, the economic growth target constraint has a positive impact on the public quality of occupational health. The effect of the economic growth target gap on the quality of public occupational health also shows a “U-shaped” trend. When the economic growth target gap is small, the quality of public occupational health deteriorates with an increase in the economic growth target gap. When the economic growth target gap is large, the quality of public occupational health improves the economic growth target gap. The above conclusions show that the impact of economic growth target setting on the quality of public occupational health is not constant. The relationship between these variables is verified at the provincial and city levels, and our conclusions are still valid after endogeneity and robustness testing.

These findings provide a valuable reference for the government to balance the relationship between economic development and occupational health. In the context of emphasising high-quality economic development, local governments need to make a full study of the economic development and occupational health condition of their jurisdictions and then combining their comparative advantages and development potential determined by their factor endowments and formulating annual economic growth targets according to local conditions. From the perspective of improving the quality of public occupational health, the assessment of GDP indicators in the underdeveloped areas should be weakened as far as possible, and the weight of safety governance should be adjusted in the assessment of officials to encourage local governments to adjust the target management centre and drive them to pay more attention to safety governance.

## Data Availability Statement

The original contributions presented in the study are included in the article, further inquiries can be directed to the corresponding author.

## Author Contributions

F-FW: conceptualisation and methodology. W-JD: writing and editing. HC: writing-original draft. QG: writing and reviewing. Z-WD: software and data preparation. H-CD: data preparation and reviewing. All authors contributed to the article and approved the submitted version.

## Funding

This research is partly supported by the Philosophy and Social Sciences Fund of Guangxi Province (20CJY001), Guangxi Higher Education Undergraduate Teaching Reform Project (2021JBG249), the Major Research Project of Philosophy and Social Science of Ministry of Education (20JZD029), the National Social Science Fund of China (17XRK002), the National Social Science Fund of China (20BJL087), and Education Planning Project of Guangxi Province (2021A043).

## Conflict of Interest

The authors declare that the research was conducted in the absence of any commercial or financial relationships that could be construed as a potential conflict of interest.

## Publisher's Note

All claims expressed in this article are solely those of the authors and do not necessarily represent those of their affiliated organizations, or those of the publisher, the editors and the reviewers. Any product that may be evaluated in this article, or claim that may be made by its manufacturer, is not guaranteed or endorsed by the publisher.

## References

[1] Tuan-Biao J Zi-Wei D Yu-Peng Z Hao C Qing G. The effect of urbanization on population health: evidence from China. Front Public Health. (2021) 9:706982. 34222193 10.3389/fpubh.2021.706982 PMC8242255

[2] Li X Liu C Weng X Zhou L-A. Target setting in tournaments: theory and evidence from China. Econ J. (2019) 129:2888–915. 10.1093/ej/uez018

[3] Du J Yi H. Target setting, political incentives, and the tricky trade-off between economic development and environmental protection. Public Administr. (2021) 12:7468. 10.1111/padm.12768

[4] Rothstein B. The Chinese paradox of high growth and low quality of government: the cadre organization meets max weber: The Chinese Paradox. Governance. (2015) 28:533–48. 10.1111/gove.12128

[5] Chen Y Li H Zhou L-A. Relative performance evaluation and the turnover of provincial leaders in China. Econ Lett. (2005) 88:421–5. 10.1016/j.econlet.2005.05.003

[6] Li H Zhou L-A. Political turnover and economic performance: the incentive role of personnel control in China. J Public Econ. (2005) 89:1743–62. 10.1016/j.jpubeco.2004.06.009

[7] Yao Y Zhang M. Subnational leaders and economic growth: evidence from Chinese cities. J Econ Growth. (2015) 20:405–36. 10.1007/s10887-015-9116-1

[8] Blanchard OJ Shleifer A. Federalism with and without political centralization. China Versus Russia. SSRN J. (2000) 76:16. 10.3386/w7616

[9] Brixiova Z. Decentralization and local governance in developing countries: a comparative perspective. Comp Econ Stud. (2008) 50:158–60. 10.1057/palgrave.ces.8100233

[10] Qian Y Weingast BR. Federalism as a Commitment to Preserving Market Incentives. Journal of Economic Perspectives. (1997) 11:83–92. 10.1257/jep.11.4.83

[11] Xu C. The fundamental institutions of China's reforms and development. J Econ Lit. (2011) 49:1076–151. 10.1257/jel.49.4.1076

[12] Zhong M Wang P Ji M Zeng X-H Wei H-X. Promote or inhibit: economic goal pressure and residents' health. Front Public Health. (2021) 9:725957. 10.3389/fpubh.2021.725957 34381757 PMC8350158

[13] Jiang J. Making bureaucracy work: patronage networks, performance incentives, and economic development in China. Am J Pol Sci. (2018) 62:982–99. 10.1111/ajps.12394

[14] Chen H Qi H Long R Zhang M. Research on 10-year tendency of China coal mine accidents and the characteristics of human factors. Saf Sci. (2012) 50:745–50. 10.1016/j.ssci.2011.08.040

[15] Wright T. The political economy of coal mine disasters in China: “your rice bowl or your life.” *China Q*. (2004) 179:629–646. 10.1017/S0305741004000517

[16] Wang S. Regulating death at coalmines: changing mode of governance in China. J Contemp China. (2006) 15:1–30. 10.1080/10670560500331658

[17] Fisman R Wang Y. The mortality cost of political connections. Rev Econ Stud. (2015) 82:1346–82. 10.1093/restud/rdv020

[18] Jia R Nie H. Decentralization, collusion, and coal mine deaths. Rev Econ Stat. (2017) 99:105–18. 10.1162/REST_a_00563

[19] Chan HS Gao J. Death versus GDP! Decoding the Fatality Indicators on Work Safety regulation in Post-Deng China. China Q. (2012) 210:355–77. 10.1017/S0305741012000379

[20] Fisman R Wang Y. The Distortionary effects of incentives in government: evidence from china's “death ceiling” program. Am Econ J Appl Econ. (2017) 9:202–18. 10.3386/w23098

[21] Shapiro SA McGarity TO. Not so paradoxical: the rationale for technology-based regulation. Duke Law J. (1991) 1991:729–52. 10.2307/1372711

[22] Jordan A Wurzel RKW Zito A. The rise of ‘new' policy instruments in comparative perspective: has governance eclipsed government? Polit Stud. (2005) 53:477–96. 10.1111/j.1467-9248.2005.00540.x

[23] Steurer R. Disentangling governance: a synoptic view of regulation by government, business and civil society. Policy Sci. (2013) 46:387–410. 10.1007/s11077-013-9177-y

[24] Latham GP Borgogni L Petitta L. Goal setting and performance management in the public sector. Int Public Manage J. (2008) 11:385–403. 10.1080/10967490802491087

[25] Bevan G Hood C. What's measured is what matters: targets and gaming in the english public health care system. Public Adm. (2006) 84:517–38. 10.1111/j.1467-9299.2006.00600.x

[26] Kelman S Friedman JN. Performance improvement and performance dysfunction: an empirical examination of distortionary impacts of the emergency room wait-time target in the English national health service. J Public Administr Res Theory. (2009) 19:917–46. 10.1093/jopart/mun028

[27] Datar S Kulp SC Lambert RA. Balancing performance measures. J Account Res. (2001) 39:75–92. 10.1111/1475-679X.00004

[28] Holmstrom B Milgrom P. Multitask principal–agent analyses: incentive contracts, asset ownership, and job design. J Law Econ Organiz. (1991) 7:24–52. 10.1093/jleo/7.special_issue.24

[29] Nie H Jiang M Wang X. The impact of political cycle: evidence from coalmine accidents in China. J Comp Econ. (2013) 41:995–1011. 10.1016/j.jce.2013.04.002

[30] Acemoglu D Johnson S Robinson J. Institutions as the fundamental cause of Long-Run growth. Handbook Econ Grow. (2005) 1:385–472. 10.1016/S1574-0684(05)01006-3

[31] Ma L. Performance feedback, government goal-setting, and aspiration level adaptation: evidence from Chinese provinces. Public Adm. (2016) 94:452–71. 10.1111/padm.12225

[32] Jie G. Political rationality vs. technical rationality in china's target-based performance measurement system: the case of social stability maintenance. Policy Soc. (2015) 34:37–48. 10.1016/j.polsoc.2015.03.005

[33] Edin M. State capacity and local agent control in China: CCP cadre management from a township perspective. China Q. (2003) 173:35–52. 10.1017/S0009443903000044

[34] Wang K-H Umar M Akram R Çaglar E. Is technological innovation making world “greener”? an evidence from changing growth story of China. Technol Forecast Soc Change. (2021) 21:120516. 10.1016/j.techfore.2020.120516

[35] Heilmann S Shin L Hofem A. National planning and local technology zones: experimental governance in China's Torch programme. China Q. (2013) 216:896–919. 10.1017/S0305741013001057

[36] Su C-W Song Y Umar M. Financial aspects of marine economic growth: From the perspective of coastal provinces and regions in China. Ocean Coast Manag. (2021) 204:105550. 10.1016/j.ocecoaman.2021.105550

[37] Comfort LK Waugh WL Cigler BA. Emergency management research and practice in public administration: emergence, evolution, expansion, and future directions. Public Adm Rev. (2012) 72:539–47. 10.1111/j.1540-6210.2012.02549.x

[38] Comfort LK Zhang H. Operational networks: adaptation to extreme events in China. Risk Anal. (2020) 40:981–1000. 10.1111/risa.13442 31943307

[39] Waugh WL Streib G. Collaboration and leadership for effective emergency management. Public Adm Rev. (2006) 66:131–40. 10.1111/j.1540-6210.2006.00673.x

[40] Lu L Li W Mead J Xu J. Managing major accident risk from a temporal and spatial perspective: a historical exploration of workplace accident risk in China. Saf Sci. (2020) 121:71–82. 10.1016/j.ssci.2019.08.035

[41] Barth A Winker R Ponocny-Seliger E Sögner L. Economic growth and the incidence of occupational injuries in Austria. Wien Klin Wochenschr. (2007) 119:158–63. 10.1007/s00508-006-0726-7 17427018

[42] Paulozzi LJ Ryan GW Espitia-Hardeman VE Xi Y. Economic development's effect on road transport-related mortality among different types of road users: A Cross-sectional international study. Acc Anal Prevent. (2007) 39:606–17. 10.1016/j.aap.2006.10.007 17092473

[43] Zhi-Ting Y Min Z Qing G Hong-Xiang W Xi-Hao Z. The impact of digital economy on residents' health: based on the perspective of population ageing. Front Public Health. (2021) 9:725971. 10.3389/fpubh.2021.725971 34381758 PMC8350039

[44] Hao C Yu-Peng Z Zi-Wei D Qing G Rui J. Crowding-out or crowding-in: government health investment and household consumption. Front Public Health. (2021) 9:706937. 10.3389/fpubh.2021.706937 34178935 PMC8226022

[45] Esarey J Schwindt-Bayer L. Women's representation, accountability and corruption in democracies. Br J Polit Sci. (2017) 48:1–32. 10.1017/S0007123416000478

[46] Stock J Yogo M. Testing for weak instruments in linear iv regression. SSRN eLibrary. (2002) 11:284. 10.3386/t0284

[47] Blundell R Bond S. Estimation with persistent panel data: an application to production functions. Econ Rev. (2000) 19:321–40. 10.1080/07474930008800475

